# Longitudinal Changes in Transfusion Practice in Myelodysplastic Syndromes: A Population Data‐Linkage Study

**DOI:** 10.1111/ejh.70233

**Published:** 2026-05-31

**Authors:** Allison Mo, Le Thi Phuong Thao, Cameron Wellard, Erica M. Wood, Jake Shortt, Zoe K. McQuilten

**Affiliations:** ^1^ Transfusion Research Unit, School of Public Health & Preventive Medicine, Faculty of Medicine, Nursing & Health Sciences Monash University Melbourne Australia; ^2^ Monash Haematology, Monash Health Melbourne Australia; ^3^ Austin Pathology and Department of Haematology Austin Health Melbourne Australia; ^4^ Department of Medicine, School of Clinical Sciences, Faculty of Medicine, Nursing & Health Sciences Monash University Melbourne Australia; ^5^ Department of Haematology Alfred Health Melbourne Australia

**Keywords:** myelodysplastic syndromes, platelet transfusion, red cell transfusion

## Abstract

**Background:**

Real‐world data on transfusion needs of patients with myelodysplastic syndromes (MDS), and the impacts of disease‐modifying therapies (DMTs) are sparse. In 2011, 5‐azacitidine became the first funded DMT for MDS and chronic myelomonocytic leukaemia (CMML) patients in Australia and national patient blood management (PBM) guidelines were published.

**Aims:**

Describe red blood cell transfusion (RBC‐T) and platelet transfusion (PLT‐T) burden in MDS/CMML and any changes since 2011.

**Method:**

Retrospective longitudinal cohort study of all MDS/CMML patients in the state of Victoria (population 6.5 million) from 2009 to 2022, linking hospital admissions dataset, cancer and death registries.

**Results:**

7043 patients (5715 MDS; 1328 CMML), with median follow‐up 1.8 years (interquartile range [IQR]: 0.5–4.0 years), had 55 048 RBC‐T and 10 749 PLT‐T episodes. By 5 years post‐diagnosis, patients had on average 14 RBC‐T and 3 PLT‐T admissions, with transfusion burden affected by MDS subtype. PLT‐T were significantly higher post‐2011 versus pre‐2011 (0.8 more PLT‐T admissions by 2 years, *p* < 0.001), with no difference in RBC‐T admissions.

**Conclusion:**

Since 2011, RBC‐T burden has remained stable, but PLT‐Ts have increased. Given the potential costs and limited PLT inventories, this impacts blood supply planning and highlights the need for research to optimise PLT‐T in MDS/CMML patients.

## 1 Introduction

Transfusion is key to supportive care management for many patients with myelodysplastic syndromes (MDS) and chronic myelomonocytic leukaemia (CMML). Approximately one‐third of MDS patients are red blood cell transfusion (RBC‐T) dependent. A similar number receive platelet transfusions (PLT‐T) [1]. In recent years, disease‐modifying therapies (DMTs) which aim to slow disease progression and reduce transfusion burden have become widely available.

Real‐world data on patients' transfusion needs can inform clinical management, empower patients to understand their likely future management pathways and enable health services and blood suppliers to project blood product needs. This includes the need for information on the potential impacts of DMTs and understanding how an individual patient's transfusion needs may change over time.

Several studies have described RBC transfusion burden in low‐risk MDS [2, 3, 4] but information on other MDS risk categories is limited and there are few data on changes in transfusion burden over time. Furthermore, there is little contemporary data on PLT‐T burdens and trends in MDS despite international trends showing an overall increase in PLT‐T [5].

In Australia, from 2011, subcutaneously administered 5‐azacitidine became the first funded DMT for patients with IPSS classification intermediate‐2 or high‐risk MDS and chronic‐myelomonocytic leukaemia‐2 (CMML2) [6]. 5‐azacitidine remains the main DMT used in Australia for non‐deletion 5q subtype MDS. National patient blood management (PBM) guidelines were also published in 2011, which advise against prophylactic PLT‐T in MDS with thrombocytopenia [7] however it is unclear whether these recommendations are being followed in the real‐world.

We aimed to describe changes in RBC‐T and PLT‐T burden in Australian MDS and CMML patients over a 14‐year period, and explore whether these were impacted by the introduction of DMTs and PBM guidelines.

## 2 Methods

Following Monash University ethics approval (HREC #18339), we conducted a retrospective cohort study of all patients ≥ 18 years of age with a diagnosis of MDS or CMML admitted to Victorian hospitals from 2009 to 2022. Ethics approval included waiver of patient consent, as the study was conducted retrospectively using de‐identified data.

### 2.1 Data Sets

Victoria is Australia's second most populous state, with a population of 6.5 million [8]. Publicly funded universal healthcare, including public hospital admission for transfusion, is available to all Australian residents. Additional private health insurance is also used by 45% of the population [9], which enables private hospital care including transfusion.

In this study, linkage of three population datasets was provided by the Victorian Department of Health were as follows:

1. Victorian Admitted Episodes Dataset (VAED): contains all statewide public and private hospital admissions data including patient demographics, diagnoses and procedures. All hospitals must report patient admission activity to the Department of Health; these data are used by the Department for funding, monitoring, health care planning and research. Data (diagnoses, procedures including transfusion) are coded by trained coders using the Australian modification of the International Statistical Classification of Disease and Related Health Problems, Tenth Revision (ICD‐10‐AM) [10] and the Australian Classification of Health Interventions (ACHI) [11]. Data quality assurance standards are maintained via regular audits [12].
2. Victorian Cancer Registry (VCR): All malignancies including MDS require mandatory notification to state‐based cancer registries.
3. Victorian Death Index (VDI): Records all deaths in Victoria, including date and cause of death, allowing identification of out‐of‐hospital mortality.

### 2.2 Search Strategy and Data Collection

All hospital admissions from 2009 to 2022 involving patients ≥ 18 years of age, which included an ICD‐10‐AM diagnostic code of MDS (D46, D460, D461, D462, D464, D465, D466, D467, D469) or CMML (C931, C9311) were extracted from VAED. For each admission, patient demographics, admission and discharge date, date of death (if during hospitalisation), diagnoses, comorbidities and procedure codes (including transfusion) were extracted. From the VCR, all new MDS/CMML diagnoses from 2009 to 2022 were extracted. Linkage of the three datasets was facilitated by the Department of Health using encrypted linkage identification numbers. Dates and causes of death were extracted from the VDI. As the VAED dataset is based upon hospital discharge summaries, to mitigate the risk of misclassification due to transcription, transcribing or coding errors within the VAED dataset, patients who did not have a confirmed MDS/CMML diagnosis in the VCR were excluded.

Hospital admissions prior to the MDS/CMML diagnosis date in the VCR were excluded.

Transfusions were identified via ACHI codes (RBC‐T:1370602, PLT‐T:1370603). In Victoria, all RBC‐T and PLT‐T occur in a hospital setting (either as a day admission for outpatients or a multi‐day inpatient admission). Thus, our study captures all transfusion episodes regardless of whether the patient is receiving transfusion in an inpatient ward or an outpatient day‐transfusion ward; each of these is counted as a single transfusion admission episode.

Patients were censored at the occurrence of acute myeloid leukaemia (AML) transformation or bone marrow transplantation, as these events will impact transfusion burden.

Comorbidities were classified according to the Charlson comorbidity index (CCI) based on ICD‐10‐AM comorbidity codes using a previously validated model [13]; the highest CCI (i.e., highest comorbidity burden) was calculated across all admissions.

MDS histologic subtypes included in the ICD‐10 AM definition include: refractory anaemia without ring sideroblasts, refractory anaemia with ring sideroblasts (RARS), refractory anaemia with excess of blasts (RAEB), refractory anaemia with multilineage dysplasia, MDS with isolated deletion 5q (del5q), other MDS and MDS unspecified. CMML diagnoses include all stages of CMML.

### 2.3 Statistical Analysis

Summary statistics are presented as *n* (%) for categorical variables and median (interquartile range, IQR) for continuous variables.

Patients were followed‐up from diagnosis until death, censoring event or end of the study.

For calculating transfusion burden over time, patients with < 1 day follow‐up were excluded as these would not significantly contribute to the overall burden. The mean cumulative function (MCF) was used to estimate the average number of transfusion episodes per patient by a given time [14] from MDS diagnosis, and compared by MDS subtype, whether admissions were ‘pre’ versus ‘post’ 2011 and the year of MDS diagnosis (to distinguish the impact of the 2011 DMT/PBM guideline changes from underlying secular trends in supportive care and transfusion practice). Variances of the MCF were estimated based on the Lawless and Nadeau method [15]. Overall survival was calculated by Kaplan–Meier estimator. All analyses were conducted using Stata version 11.0 (Statacorp LP, College Station, Texas) or R version 4.4.2 [16].

## 3 Results

### 3.1 Patient Characteristics

**FIGURE 1:**
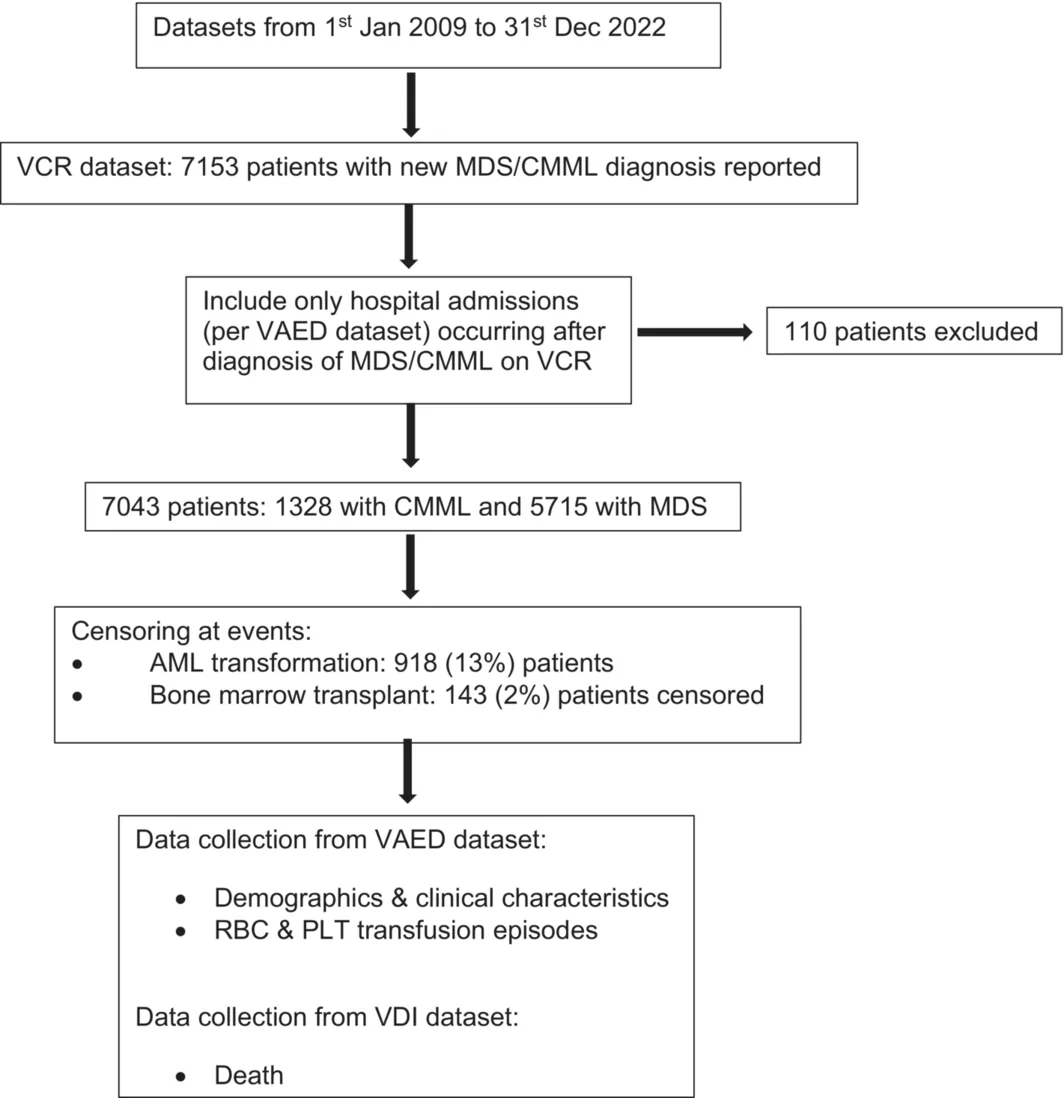
Flow diagram of study participants.

From 1 January 2009 to 31 December 2022, 7153 patients with a new diagnosis of MDS/CMML were reported to the VCR. There were 169 523 hospital admissions involving 12 070 patients with a diagnostic code for MDS/CMML recorded in the VAED. After combining these datasets and including only hospital admissions occurring after a confirmed diagnosis of MDS/CMML on the VCR, this resulted in a dataset of 7043 included patients. Of these, 918 patients were censored at AML transformation and 143 patients at bone marrow transplantation. (Figure 1).

The median follow‐up time, from diagnosis until death or censoring event or end of study, was 1.8 years (IQR: 0.5–4.0 years). During the study period, there were 55 048 RBC‐T and 10 479 PLT‐T admission episodes. 4771 patients (67.7%) died.

Patient demographics are shown in Table 1. Patients were predominantly male (*n* = 4242, 60.2%); 78.4% were ≥ 70 years of age at diagnosis. 1328 patients (18.9%) had CMML and 5715 (81.1%) had MDS. The commonest MDS classifications were MDS unspecified (34.5%), refractory anaemia with multilineage dysplasia (20.2%) and RAEB (12.8%). Over half of patients had a low burden of co‐morbidities, defined as maximum CCI ≤ 2 (*n* = 3634, 52.4%), but 20.6% had a high CCI ≥ 6.

**TABLE 1:** Patient clinical characteristics.

Characteristic	*N* (%) (*N* = 7043)
Gender	
Male	4242 (60.2%)
Female	2698 (38.3%)
Unknown	103 (1.5%)
Age groups at MDS/CMML diagnosis	
18–24	17 (0.24%)
25–29	19 (0.27%)
30–34	19 (0.27%)
35–39	29 (0.41%)
49–44	45 (0.64%)
45–49	55 (0.78%)
50–54	100 (1.42%)
55–59	200 (2.84%)
60–64	389 (5.52%)
65–69	648 (9.20%)
70–74	943 (13.39%)
75–79	1277 (18.13%)
80–84	1430 (20.30%)
85+	1872 (26.58%)
Disease subtypes^a^	
CMML	1328 (18.9%)
Refractory anaemia without ring sideroblasts	396 (5.6%)
Refractory anaemia with ring sideroblasts	272 (3.9%)
Refractory anaemia with excess of blasts (RAEB)	901 (12.8%)
Refractory anaemia with multilineage dysplasia	1423 (20.2%)
MDS with isolated del(5q)	69 (1.0%)
Other MDS	223 (3.17%)
MDS unspecified	2431 (34.5%)
Charlson comorbidity index (CCI) score^b^	
0–2	3634 (52.4%)
3–5	1875 (27.0%)
6+	1431 (20.6%)
RBC transfusions	
Total RBC transfusion admissions	55 048
No. (%) of patients who received RBC transfusion	4574 (65%)
Platelet transfusions	
Total platelet transfusion admission	10 749
No. (%) of patients who received platelet transfusion	1825 (26.1%)

^a^ Subtype classification based upon ICD‐10‐AM diagnoses in VCR.

^b^ Worst CCI across all admissions.

There was a stable rate of new MDS and CMML diagnoses over time, with a median of 468 (IQR: 447–500) new cases per year. (Figure S1).

### 3.2 Overall Survival (OS) and Transfusion Admissions

6797 patients had > 1 day follow‐up time and were included for analyses of transfusion burden over time and OS. Of the remaining 246 patients who had < 1 day follow‐up time (due to death, censoring or end of study period), only 16 had a transfusion episode.

Median OS was 3.6 years (95% CI: 3.4–3.8 years). Table 2 shows the average RBC and platelet transfusion burden over time. By one‐year post‐diagnosis, patients had on average 3.6 RBC and 0.9 platelet transfusion admissions, increasing to 14 RBC and 2.7 platelet transfusion admissions by 5 years.

**TABLE 2:** Overall survival and cumulative average RBC‐T and PLT‐T admissions per patient at 1, 3 and 5 years post diagnosis.

	Overall survival in years (median, 95% CI)	RBC‐T admissions per patient (mean, 95% CI)			PLT‐T admissions per patient (mean, 95% CI)		
		1 year post‐diagnosis	3 years post‐diagnosis	5 years post‐diagnosis	1 year post‐ diagnosis	3 years post‐diagnosis	5 years post‐diagnosis
All patients	3.6	3.6	9.3	14.0	0.9	2.0	2.7
	(3.4–3.8)	(3.4–3.7)	(8.8–9.8)	(13.1–14.8)	(0.8–1.0)	(1.7–2.3)	(2.2–3.2)
Disease subtype							
CMML	4.0	1.6	4.3	7.1	0.5	1.1	1.5
	(3.5–4.5)	(1.4–1.9)	(3.6–5.0)	(5.9–8.4)	(0.3–0.6)	(0.8–1.3)	(1.2–1.9)
Refractory anaemia without ring sideroblasts	5.9	3.1	7.5	10.3	0.7	2.4	3.1
	(4.3–6.8)	(2.4–3.8)	(5.6–9.4)	(7.6–12.9)	(0.1–1.3)	(0.06–4.8)	(0–6.3)
Refractory anaemia with ring sideroblasts (RARS)	6.7	3.6	11.8	22.4	0.2	0.3	0.6
	(5.6–8.2)	(2.9–4.4)	(9.3–14.3)	(17.5–27.4)	(0–0.5)	(0.1–0.6)	(0.2–1.0)
Refractory anaemia with excess of blasts (RAEB)	2.9	7.2	16.2	20.8	2.7	5.9	6.7
	(2.5–3.4)	(6.4–7.9)	(14.2–18.1)	(17.6–23.9)	(2.1–3.3)	(4.3–7.4)	(5.1–8.3)
Refractory anaemia with multilineage dysplasia	4.4	3.3	9.8	14.6	0.8	2.4	3.6
	(4.0–4.9)	(3.0–3.7)	(8.6–10.9)	(12.8–16.4)	(0.6–1.0)	(1.6–3.2)	(2.0–5.1)
Deletion 5q	5.8	4.5	10.0	12.7	0.7	1.7	2.2
	(4.0‐NR)	(2.9–6.5)	(6.4–13.6)	(8.4–16.9)	(0–1.4)	(0.2–3.2)	(0.5–3.8)
Other MDS	5.2	3.7	9.7	14.2	0.8	1.8	3.0
	(4.1‐NR)	(2.9–4.6)	(7.2–12.1)	(10.0–18.4)	(0.3–1.3)	(0.7–3.0)	(0.6–5.5)
MDS, unspecified	2.5	3.7	9.7	14.6	0.6	1.3	1.9
	(2.3–2.8)	(3.4–3.9)	(8.9–10.6)	(13.2–16.1)	(0.5–0.8)	(1.0–1.7)	(1.5–2.4)

Abbreviation: NR, not reached.

**FIGURE 2:**
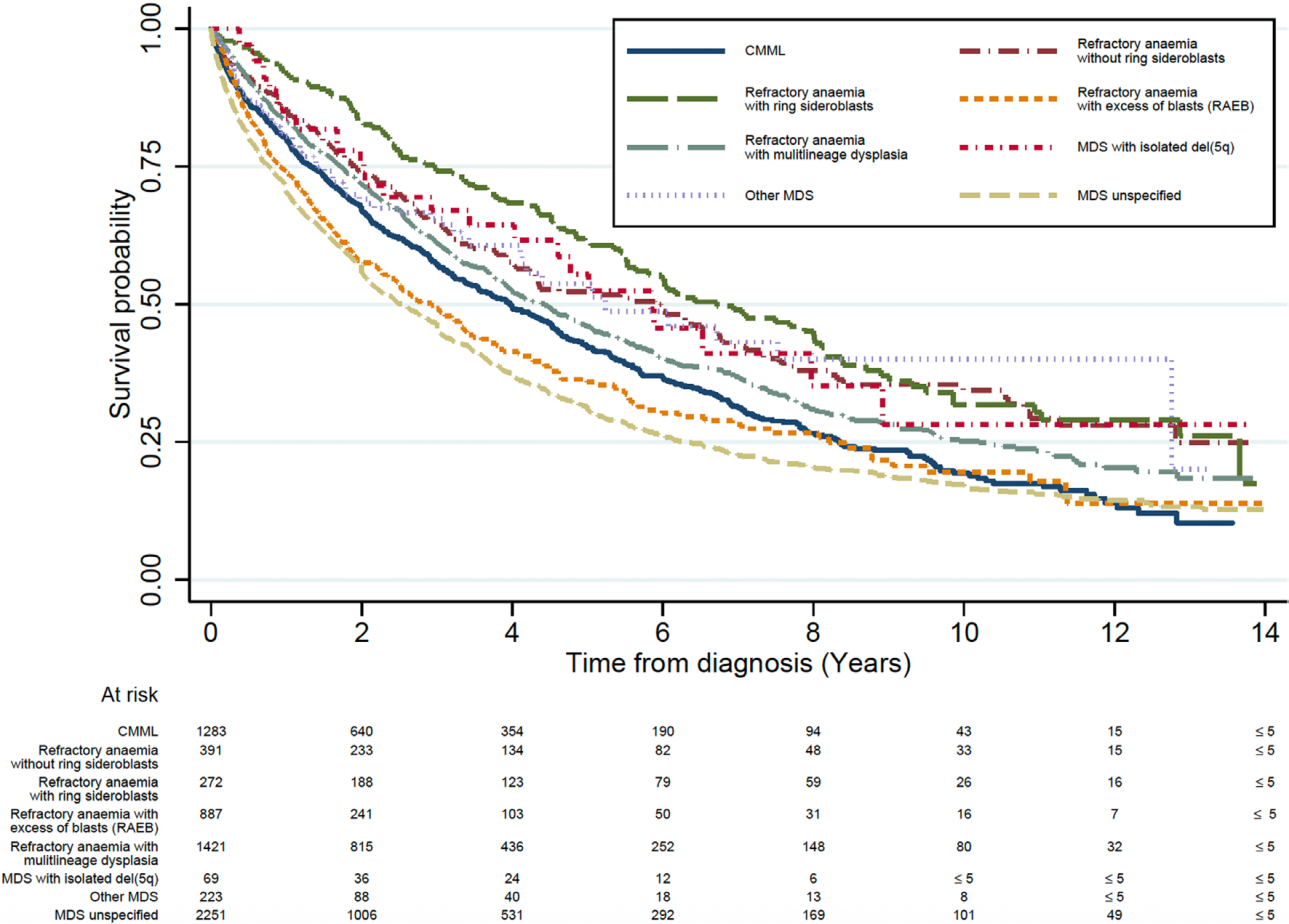
Overall survival by subtype. Shows overall survival by disease subtype. Note that, as this relates to the calculation of transfusion burden over time, patients with < 1 day follow‐up were excluded as these would not significantly contribute to the overall transfusion burden. Abbreviations: CMML, chronic myelomonocytic leukaemia; MDS, myelodysplastic syndromes.

Disease subtype significantly impacted transfusion burden and OS (Table 2, Figure 2). Patients with RARS and RAEB had the most RBC‐T admissions (22.4 and 20.8 by 5 years), with median overall survival of 6.7 and 2.9 years, respectively. Patients with CMML had the lowest RBC‐T burden (7.1 admissions by 5 years). Platelet transfusion admissions were highest for patients with RAEB (6.7 by 5 years).

**FIGURE 3:**
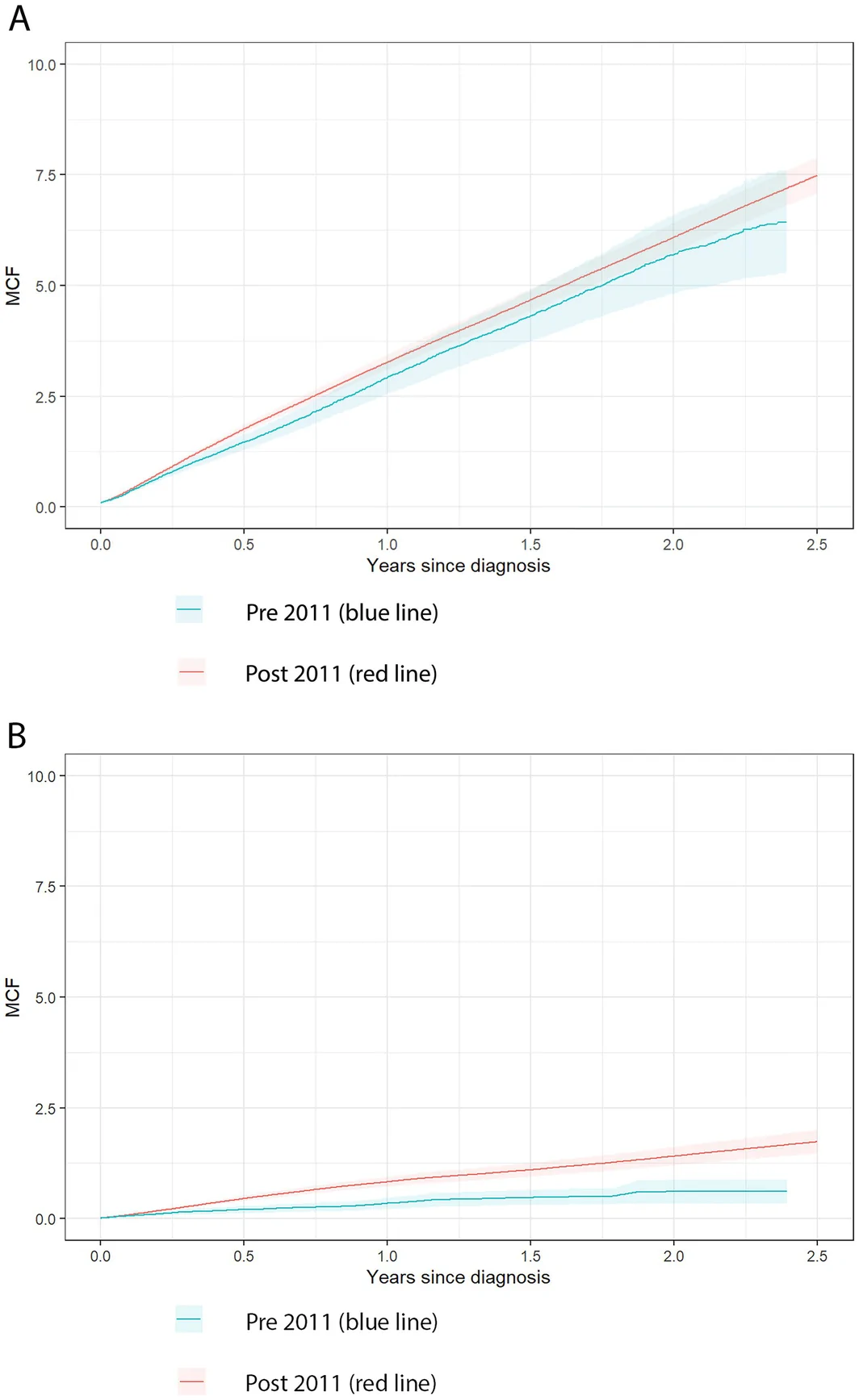
Average cumulative RBC and platelet transfusion burden over time per patient, pre‐ versus post‐2011. (A) RBC transfusion burden, comparing pre‐ versus post‐2011. MCF, mean cumulative function, which estimates the average number of transfusion episodes per patient by a given time. Comparing pre‐2011 versus post‐2011, there was no difference in RBC‐T admissions per patient over time. (B) Platelet transfusion burden, comparing pre‐ versus post‐2011. MCF, mean cumulative function, which estimates the average number of transfusion episodes per patient by a given time. Comparing pre‐2011 versus post‐2011, platelet transfusion burden was significantly higher post‐2011.

Figure 3 shows that, comparing pre‐2011 versus post‐2011, PLT‐T were significantly higher post‐2011 (0.8 more platelet transfusion admissions by 2 years compared to pre‐2011, *p* < 0.001) (Figure 3, panel B). RBC‐T admissions per patient were similar pre‐2011 versus post‐2011. (Figure 3, panel A).

There was no difference in RBC‐T or PLT‐T admissions per patient based upon year of MDS diagnosis, noting that patients diagnosed prior to 2011 who survived contributed to transfusion events in the post‐2011 period.

## 4 Discussion

This large population‐based study of Australian MDS patients shows that both RBC and platelet transfusion burden increases over individual patients' disease course and are affected by MDS subtype. On average, an MDS patient will have 14 RBC‐T and 2.7 PLT‐T admissions by 5 years post‐diagnosis. Further, PLT‐T burden has increased on a population level over time.

Our study advances the current limited understanding of transfusion burden in patients with MDS. Previous studies have largely focused on RBC‐T in specific MDS subtypes, such as low‐risk MDS [2, 3, 4]. Of note, a previous Dutch study of 292 unselected MDS patients showed that 46.6% had high transfusion burden (≥ 8 units/16 weeks) and 5.8% had low transfusion burden (3–7 units/16 weeks) [17]. However, transfusion burden was reported as a static measurement (assessing only the period with the highest transfusion burden) [17]. A Swedish study of 2311 patients showed RBC and platelet transfusion burden in the first 4 years following diagnosis varied depending on IPSS‐R stratification, but did not compare over different time periods [18]. Our study, of the largest cohort to date, adds to this existing knowledge by describing how RBC‐T and PLT‐T burden change over both a patient's MDS disease course and more broadly over time.

These findings can help patients and clinicians to anticipate future transfusion needs. Although transfusion aims to improve quality of life, the transfusion process itself may also be burdensome and impact patients' daily lives. Patients have previously reported feeling inadequately informed about the transfusion process, that time commitments (including travel time) required for transfusion negatively impact their quality of life and economic hardship due to regular transfusions [19]. Accurate information about future potential transfusion needs can empower patients to be better prepared, from the time of MDS diagnosis.

It is also important for health services to be able to forecast future blood supply needs and predict the potential impacts of DMTs and other mering therapies. In many countries, including Australia, the incidence of MDS is rising in the setting of an ageing population [20]. Given our findings of significantly increased PLT‐T burden in the 5‐azacitidine era, it is imperative that health services review and plan for potential ongoing increases in platelet use. Platelets have a short shelf life, limited availability and are costly.

The reason for the increase in PLT‐T burden since 2011 is unclear. It may reflect increased use of DMTs which often cause thrombocytopenia (Grade 3–4 thrombocytopenia occurs in one‐third of patients on 5‐azacitidine, predominantly within the first 4 cycles) [21] and heightened concerns about bleeding. However, their impact on bleeding is uncertain, with large studies showing no difference versus best supportive care [22] (0.56 vs. 0.60 Grade 3–4 bleeding events per patient‐year) [23]. There is also clinical uncertainty and no published randomised clinical trial data on the efficacy and safety of regular prophylactic PLT‐T in this setting [24]. Although PBM guidelines recommend against regular prophylactic PLT‐T, including in patients on DMTs [25] due to the lack of underlying evidence of their efficacy and safety in this context, international surveys consistently show that clinicians administer PLT‐T in MDS patients contrary to such guidelines [26, 27]. Our Australasian survey showed a greater tendency for clinicians to give prophylactic PLT‐T once patients started 5‐azacitidine [26]. This practice variation may be due to the lack of other effective treatments for MDS‐related thrombocytopenia.

Our study has some limitations, mainly due to limitations of the included datasets. First, medication data is not recorded in state‐based population databases, thus we did not have specific information on the use of 5‐azacitidine or other DMTs in individual patients. However, these have been widely used in Australia in eligible MDS and CMML patients since 2011. An analysis of a 10% random sample of Australians between 2016 and 2021 identified 351 patients nationally who initiated 5‐azacitidine [28], implying roughly 3500 patients nationally. Over a similar period, publicly available data reported 11 441 new diagnoses of MDS and CMML nationally [29], thus suggesting 5‐azacitidine was used in ~30% of cases (noting that some treatments may have been for patients with earlier diagnoses who later progressed, and the time periods do not align perfectly).

Further, we were not able to differentiate patients into IPSS‐R categories as the datasets do not include diagnostic bone marrow, cytogenetics data or molecular data and these could not be incorporated into the analysis. Molecular data was not available for the earlier periods of the study in any case, reflecting the evolving nature of diagnostic work‐up over time. This limits assessment of transfusion requirements according to disease biology; however, our primary aim was to describe real‐world transfusion burden on a population level. Transfusion admissions were identified via ACHI codes but these do not include the number of individual RBC or platelet units transfused at each admission. The reason for transfusion (e.g., prophylactic versus therapeutic PLT‐T for bleeding) was unknown as this information is not available in administrative datasets; thus our findings reflect overall utilisation and transfusion burden rather than indication‐specific practice patterns.

The retrospective design and geographically restricted cohort may limit the generalisability; however, they still provide important real‐world insights given the limited contemporary data from our region. We also acknowledge that we could not fully account for other supportive care interventions, nor could we separate the potential impacts of the introduction of 5‐azacitidine alongside PBM guidelines, which both occurred in the same period. Our findings therefore should be interpreted as descriptive of temporal practice patterns. Nevertheless, there are limited other supportive care options in our setting, as erythropoiesis‐stimulating agents (ESAs) and erythroid maturation agents (EMA) are not reimbursed for MDS‐related anaemia in Australia, and there are no funded therapies for MDS‐related thrombocytopenia. While regional differences in supportive care exist, transfusion rates in MDS patients remain high even in countries where ESAs/EMAs are widely available [19] thus suggesting broader relevance of our findings. Given that supportive care options for MDS‐related thrombocytopenia remain limited globally [30], our observed platelet transfusion patterns may be informative across different healthcare systems. Prospective and multicentre international studies across diverse regions will be valuable to confirm these findings.

Strengths of this study include that this is the largest of its kind to describe both RBC and platelet transfusion burden in a broad group of MDS and CMML patients, with information relevant on an individual patient level and population level. Notably, the increased platelet transfusion requirements quantifies an ongoing supportive care burden and highlights an unmet need in thrombocytopenia management. Although our findings may not immediately change MDS management paradigms directly, they provide useful information for individual patients and clinicians, can inform resource planning, underscore the limitations of existing supportive strategies for thrombocytopenia, and the rationale for evaluating emerging treatments and optimising transfusion practices.

Our findings have implications for future research. Recent international guidelines do not provide specific guidance on the use of routine prophylactic platelet transfusions in patients with hypoproliferative thrombocytopenia who are not undergoing chemotherapy or allogeneic bone marrow transplant [31]. This is largely due to a lack of evidence of efficacy and concerns about potential harms; there have been no completed clinical trials in this area and only one unreported trial, terminated due to poor recruitment [32]. Given the increasing demand for platelet transfusions, there is a pressing need for clinical trials evaluating the effectiveness of platelet transfusion or other treatments for MDS‐related thrombocytopenia.

## 5 Conclusion

RBC and platelet transfusion burden in MDS/CMML patients are associated with disease subtype and increase over the disease course. Since 2011, although RBC transfusion burden has remained stable, platelet transfusion burden has significantly increased. Further research is needed to understand why this has occurred, and if it may be related to increasing use of disease‐modifying therapies such as 5‐azacitidine since 2011. Given the costs and limited supply of platelet products, our findings have implications for national blood supply planning, future guideline development and highlight the need for research to optimise platelet transfusion, ensuring they are used appropriately and effectively.

## Author Contributions

A.M., E.M.W., J.S. and Z.K.M. designed the study; A.M., L.T.P.T., C.W. and Z.K.M. did the data analysis; A.M., L.P.T.P., C.W., E.M.W., J.S. and Z.K.M. wrote the paper.

## Funding

This project received research grant funding from the Australian and New Zealand Society of Blood Transfusion (ANZSBT). A.M. receives PhD scholarship funding from the National Health and Medical Research Council (NHMRC, grant no. 1151512), National Blood Authority, Monash University, and Haematology Society of Australia and New Zealand. J.S. and Z.K.M. are supported by Australian NHMRC Emerging Leadership Fellowships (grant no. Z.K.M.: 1194811, J.S.: 2009177). EW is supported by an NHMRC Leadership Fellowship (grant no. 1177784). AML and MDS research at Monash Haematology are supported by a donation from Dr. Alexander Baxter.

## Ethics Statement

This study was received Monash University ethics approval (HREC #18339).

## Consent

This study received ethics review board approval for waiver of patient consent, as it was conducted retrospectively and all patient data were de‐identified.

## Conflicts of Interest

J.S. has served on advisory boards for Bristol Myers Squibb, Otsuka and Novartis. J.S. has received research funding to their institution from Astex Pharmaceuticals. J.S. has received speaker's fees to their institution from Novartis (all disclosures for J.S. are outside of the submitted work). E.W. and Z.M. have received research funding to their institution from: Abbvie, Amgen, Antengene, AstraZeneca, Beigene, Bristol‐Myers Squibb, CSL Behring, Gilead, GSK, Janssen‐Cilag, Novartis, Pfizer, Roche, Sanofi and Takeda, and research support from Sobi, all outside the submitted work. All other authors declare no conflicts of interest.

## Supporting information


**Figure S1:** MDS and CMML diagnoses over time.

## Data Availability

The data that support the findings of this study are not publicly available due to privacy considerations. Access to the underlying dataset is controlled by the Victorian Department of Health.
