# Supplementary material for: Longitudinal Changes in Transfusion Practice in Myelodysplastic Syndromes: A Population Data‐Linkage Study
Source: Eur J Haematol. 2026 May 31;117(4):1007–15. doi: 10.1111/ejh.70233 (PMC13542939; doi:10.1111/ejh.70233)
Supplement: Supplementary file 1 [file EJH-117-1007-s001.docx]

Supplementary figure 1: MDS and CMML diagnoses over time

N of patients

| Year | N of MDS diagnoses |
| --- | --- |
| 2009 | 452 |
| 2010 | 441 |
| 2011 | 468 |
| 2012 | 468 |
| 2013 | 440 |
| 2014 | 498 |
| 2015 | 445 |
| 2016 | 415 |
| 2017 | 452 |
| 2018 | 532 |
| 2019 | 509 |
| 2020 | 564 |
| 2021 | 501 |
| 2022 | 481 |
